# Influence of pyrolysis temperature on lead immobilization by chemically modified coconut fiber-derived biochars in aqueous environments

**DOI:** 10.1007/s11356-016-7428-0

**Published:** 2016-08-29

**Authors:** Weidong Wu, Jianhong Li, Nabeel Khan Niazi, Karin Müller, Yingchao Chu, Lingling Zhang, Guodong Yuan, Kouping Lu, Zhaoliang Song, Hailong Wang

**Affiliations:** 1 Ministry of Education Key Laboratory of Protection and Development Utilization of Tropical Crop Germplasm Resources, Hainan University, Haikou, 570228 China; 2 Key Laboratory of Soil Contamination Bioremediation of Zhejiang Province, Zhejiang A & F University, Lin’an, Hangzhou 311300 China; 3 Institute of Soil and Environmental Sciences, University of Agriculture Faisalabad, Faisalabad, 38040 Pakistan; 4 MARUM and Department of Geosciences, University of Bremen, Bremen, D-28359 Germany; 5 The New Zealand Institute for Plant & Food Research Limited, Ruakura Research Centre, Private Bag 3123, Hamilton, 3240 New Zealand; 6 School of Environmental and Chemical Engineering, Foshan University, Foshan, Guangdong 528000 China; 7 Guangdong Dazhong Agriculture Science Co. Ltd., Hongmei Town, Dongguan City, Guangdong 523169 China

**Keywords:** Coconut fibers, Biochar, Modified biochar, Functional group, Wastewater treatment, FTIR, SEM

## Abstract

Biochar has received widespread attention as an eco-friendly and efficient material for immobilization of toxic heavy metals in aqueous environments. In the present study, three types of coconut fiber-derived biochars were obtained by pyrolyzing at three temperatures, i.e., 300, 500, and 700 °C. In addition, nine types of biochars were prepared by chemical modification with ammonia, hydrogen peroxide, and nitric acid, respectively, which were used to investigate changes in physico-chemical properties by inter alia, Fourier transformation infrared spectrophotometry (FTIR), scanning electron microscope (SEM), and BET specific surface area analysis. Batch sorption experiments were carried out to determine the sorption capacity of the biochars for lead (Pb) in aqueous solutions. Results showed that the cation exchange capacity of biochar pyrolyzed at 300 °C and modified with nitric acid increased threefold compared to the control. Loosely corrugated carbon surface and uneven carbon surface of the biochar pyrolyzed at 300 °C were produced during ammonia and nitric acid modifications. Removal rate of Pb by the coconut biochar pyrolyzed at 300 °C and modified with ammonia was increased from 71.8 to 99.6 % compared to the untreated biochar in aqueous solutions containing 100 mg L^−1^ Pb. However, chemical modification did not enhance adsorption of Pb of the biochars pyrolyzed at higher temperatures (e.g., 500 or 700 °C), indicating that resistance of biochars to chemical treatment increased with pyrolysis temperature.

**Electronic supplementary material:**

The online version of this article (doi:10.1007/s11356-016-7428-0) contains supplementary material, which is available to authorized users.

## Introduction

Biochar is a carbon (C)-rich material produced by thermal decomposition of biomass in an oxygen-limited environment (Al-Wabel et al. 2013). Biochar typically has a high sorption capacity for contaminants, and on a long-term basis, it may also sequester C (Zhang et al. 2014; Dong et al. 2015). Due to its large specific surface area (SSA), a variety of surface functional groups (hydroxyl, phenolic, carboxyl), microporous structure, high cation exchange capacity (CEC), and high pH, biochar is often regarded as an environmentally friendly and efficient sorbent for immobilizing heavy metals in aqueous environments and/or in pore water of soils and sediments (Zhang et al. 2013; Yang et al. 2016a, b). Many previous studies have reported high sorption capacities of biochars for different pollutants (Mohan et al. 2007; Keiluweit et al. 2010). Of all heavy metals, lead (Pb) is of major concern. Due to its wide use, Pb has been contaminating many different environments ranging from wastewater to soil.

The surface chemical composition and the physico-chemical properties of biochar can be chemically modified, which could greatly influence its sorption capability for heavy metals as well as its stability in aqueous environments (Chen 2012; Liu et al. 2013; Huff and Lee 2016). Although biochar has been used to remove heavy metals from aqueous solutions, limited research has been directed to explore the influence of various chemical modification methods on the heavy metal removal capacity of biochars produced in a range of pyrolysis temperatures. The basic properties of biochars may differ from chemically modified biochars because of potential dissolution of soluble mineral oxides and chemical reactions of functional groups on biochars during the different modification treatments. Because of the higher surface area and the larger pore volume of oak bark char compared to pine wood char, oak bark char offers a greater potential for Pb, cadmium (Cd), and arsenic (As) sorption than pine wood char (Mohan et al. 2007). It was reported that the CEC of a pinewood-derived biochar was increased by 75 % after modification with hydrogen peroxide (H_2_O_2_) owing to the increased number of oxygen-containing functional groups (Huff and Lee 2016).

Several organic waste materials have been reported to be good feedstocks for producing biochar (Wang et al. 2010; Xu et al. 2013; Yang et al. 2016b). These have been successfully applied for removal of heavy metals from aqueous environment (Mohan et al. 2007; Xu et al. 2013). However, to our understanding, the potential of coconut fiber (CF)-derived biochar to remove heavy metals has received less attention. CF is a cellulosic material obtained from the fibrous mesocarp of coconut, which consists of about 50 % of the nut and is available in large quantities as waste product of coconut orchards in tropical areas of Asian countries, such as the Philippines, Indonesia, Malaysia, and China. For example, more than 250 million coconuts are produced annually from plantations of approximately 40,000 ha in Hainan Island, China (Wang et al. 2015). Only small amount of coconut industrial byproducts are utilized for production of activated carbon or handcrafts. CF has also been used as sorbent to remove contaminants from wastewater (e.g., Loffredo et al. 2014; Henryk et al. 2016). The performance of coconut-derived char to remove Cr from aqueous phase was investigated by Shen et al. (2012). To the best of our knowledge, research on the potential of chemical-modified coconut fiber-derived biochar (MCFB) to remove Pb in aqueous environments is scarce.

Therefore, the objectives of this study were to (1) investigate the surface chemical composition and changes of physico-chemical properties of biochars after various chemical modification methods, and (2) assess the potential use of coconut fiber biochars (CFBs) and MCFBs for removal of Pb in aqueous environments.

## Materials and methods

### Production of biochars

Coconut (*Cocos nucifera* L.) was collected from a coconut grove in the eastern suburbs of Wenchang (110.9°E, 19.6°N), Hainan Province, China. The CF was separated from the coconut flesh and water, chopped into cubes of about 1 cm × 1 cm (length × height), air-dried at room temperature (25 °C) to a moisture content of approximately 7–8 %. Air-dried cubes were placed in ceramic crucibles, covered with lids and pyrolyzed at 300, 500, and 700 °C under oxygen-limited conditions in an SX210-12 muffle furnace (Longkou Xian Ke Electricity Furnace Inc., Shandong, China) with a heating rate of approximately 20 °C per min (Yuan et al. 2011). Each of the peak temperature was maintained for 4 h before cooling to ambient temperature.

The biochars produced at different pyrolysis temperatures were grounded and passed through a 2-mm sieve. The biochars produced at 300, 500, and 700 °C were washed with deionized water and dried at 60 °C for 48 h to minimize the impact of water-soluble inorganic minerals and ash contents present on the surface of the biochar in the aqueous solutions. Biochars were stored in air-tight plastic sample bags prior to batch sorption experiments and spectroscopic and microscopic analyses. The CFBs were controls in our experiments and herein referred to as CFB300, CFB500, and CFB700, for 300, 500, and 700 °C, respectively.

For preparing the chemically modified biochars, we adapted the procedure developed in previous studies (Chen 2012; Liu et al. 2013; Huff and Lee 2016). The controls of CFBs were mixed at a 1:10 (*w*/*v*) ratio with (a) 5 % ammonia and shaken in a constant temperature water bath at 50 °C for 9 h, (b) 5 % hydrogen peroxide and shaken at 25 °C for 8 h, and (c) 2 M nitric acid shaken at 30 °C for 8 h. Biochars were washed with deionized water to remove excess chemical reagents, dried at 60 °C for 48 h, and stored in air-tight plastic sample bags prior to use in the experiments. The chemically modified biochars are hereafter collectively referred to as modified MCFBs. The biochars produced at 300, 500, and 700 °C treated with ammonia, hydrogen peroxide, and nitric acid aqueous solution are referred to as MCFB300_NH3•H2O_, MCFB500_NH3•H2O_, MCFB700_NH3•H2O_, MCFB300_H2O2_, MCFB500_H2O2_, MCFB700_H2O2_, MCFB300_HNO3_, MCFB500_HNO3_, and MCFB700_HNO3_, respectively.

### Characterization of biochars

The ash content of the biochars was determined according to the American Society for Testing and Materials (ASTM) method (D1762-84 Standard 2007). The biochar’s pH value was measured in a 1:20 (*w*/*v*) biochar to water suspension after stirring this mixture for 1 h. CEC of the CFBs and MCFBs was determined following the 1 M ammonium acetate (pH 7) method (Lu 1999). The contents of acidic and basic functional groups of CFBs and MCFBs were determined by the Boehm titration method (Wu et al. 2012). The total C, nitrogen (N), and hydrogen (H) contents were measured using a Vario EL III (Elementar Company, Germany). The oxygen (O) content was determined by difference assuming that the biochar was composed only of C, N, H, and O (Wu et al. 2012). The SSA of the biochars was determined by N_2_ adsorption isotherms applying the BET equation in the relative pressure (P/P_0_) range between 0.05 and 0.35, and taking the average area occupied by a molecule of N_2_ in the completed monolayer to be equal to 16.2 Å^2^.

### Analysis of the micromorphology using scanning electron microscope

The micromorphology of the biochars was examined using a scanning electron microscope (SEM). A biochar sample was coated with a thin layer of gold and mounted on a copper slab using a double-stick carbon tape prior to scanning it by an electron microscope model (S-3000N, Hitachi Company, Japan).

### Characterization of surface functional groups using FTIR spectroscopy

For characterizing the surface functional groups on the biochar, Fourier transformation infrared (FTIR) spectroscopy was employed. Approximately 1 mg of the finely grounded biochar was gently mixed with 200 mg of oven-dried (105–110 °C) KBr. The mixture was then pressed into a pellet for FTIR analysis. The FTIR spectrum of biochars with and without Pb adsorbed was recorded using a TENSOR 27 FTIR spectrophotometer (Bruker Company, Germany) scanning from 4000 to 500 cm^−1^ (wavenumber) at a resolution of 4 cm^−1^.

### Batch sorption experiments

Batch sorption experiments were carried out to determine the sorption capacity of CFBs and MCFBs for Pb in aqueous environments. We prepared a 100 mg L^−1^ Pb solution containing 0.01 M NaNO_3_ as background electrolyte at pH 6 to simulate Pb-contaminated wastewater. This concentration was chosen to provide maximum surface coverage of Pb on the biochar surface plus to allow the detection of surface-sorbed Pb using SEM and FTIR as described above.

The CFBs and MCFBs (0.05 g) were added into 25 mL Pb solution in 50 mL of the stock plastic centrifuge tubes and shaken for 24 h on a rotating shaker at 220 rpm and 25 °C. This time was selected based on previous studies (e.g., Lu et al. 2012).

The aqueous solutions were filtered through a 0.45-μm cellulose acetate micromembrane filter, and the concentration of Pb in the filtrate was analyzed by atomic absorption spectrophotometer (AAS). The sorption experiment was carried out in triplicate. Residual standard deviation for the AAS measurements was <2 %. In addition, Pb solutions of known concentrations were analyzed between samples to check the analytical accuracy.

### Statistical analyses

Results are expressed on a dry biochar basis and are shown as mean of three replicates per treatment. The differences of basic properties across different biochars were compared by a factorial analysis of variance (ANOVA) at a 5 % significance level (Duncan’s multiple range test). Statistical analyses were performed using SAS (version 9.1, SAS Institute Inc., USA). The FTIR spectra were pretreated using Origin software (version 9.0, OriginLab, USA).

## Results and discussion

### Physico-chemical properties of CFBs and MCFBs

The basic properties of CFBs and MCFBs produced at three temperatures are shown in Table 1. The properties of the CFBs were significantly (*P* < 0.05) affected by pyrolysis temperature. The ash contents of the CFBs produced at 300, 500, and 700 °C were 3.8, 4.9, and 6.7 %, respectively, and tended to increase with increasing pyrolysis temperature as reported earlier (Cao and Harris 2010). The ash contents of the CFBs were lower than those of biochars derived from woody feedstock (ash content 2–8 %) and rice straw (20–30 %) reported by other researchers (Keiluweit et al. 2010; Wu et al. 2012). This was ascribed to the fact that coconut fiber contains significant amounts of cellulose and hemicelluloses, which is easily decomposed at high temperatures with small residual ash amounts (Shen et al. 2012).

**Table 1:** The cation exchange capacity (CEC), pH, ash content, specific surface area (BET), and functional groups of the coconut fiber-derived biochars (CFBs) and the chemically modified coconut fiber-derived biochars (MCFBs) used in this study

Sample	pH	Ash content	CEC	Alkaline groups(mmol g^−1^)	Carboxylic acid	Weak acid ester	Phenolic hydroxyl	BET
ID		(%)	(cmol_c_ kg^−1^)		(mmol g^−1^)	(mmol g^−1^)	(mmol g^−1^)	(m^2^ g^−1^)
CFB300	7.41 ± 0.06g	3.76 ± 0.18c	72.86 ± 1.38fg	0.42 ± 0.002f	0.32 ± 0.002f	0.39 ± 0.005c	0.30 ± 0.003a	4.495
MCFB300_H2O2_	7.12 ± 0.03h	2.72 ± 0.11f	117.38 ± 2.55c	0.34 ± 0.001g	0.51 ± 0.001c	0.55 ± 0.004a	0.07 ± 0.009g	5.367
MCFB300_NH3•H2O_	8.33 ± 0.04f	2.70 ± 0.14f	106.21 ± 2.90d	0.62 ± 0.003e	0.32 ± 0.008f	0.48 ± 0.009b	0.18 ± 0.003b	8.856
MCFB300_HNO3_	3.66 ± 0.04i	1.00 ± 0.12i	298.91 ± 5.10a	0.23 ± 0.005i	1.04 ± 0.006a	0.09 ± 0.004h	0.01 ± 0.003i	4.044
CFB500	10.26 ± 0.01b	4.89 ± 0.23b	81.17 ± 3.70e	0.66 ± 0.004c	0.22 ± 0.005i	0.10 ± 0.009g	0.09 ± 0.002e	6.844
MCFB500_H2O2_	9.26 ± 0.05e	3.12 ± 0.16e	126.06 ± 2.64b	0.67 ± 0.002b	0.35 ± 0.005e	0.36 ± 0.004d	0.16 ± 0.007c	7.326
MCFB500_NH3•H2O_	9.29 ± 0.05e	3.45 ± 0.22d	84.15 ± 3.77e	0.67 ± 0.003b	0.25 ± 0.005g	0.16 ± 0.001f	0.15 ± 0.001c	24.397
MCFB500_HNO3_	3.33 ± 0.04j	1.72 ± 0.05g	102.54 ± 2.56d	0.21 ± 0.002j	0.59 ± 0.002b	0.39 ± 0.002c	0.04 ± 0.005h	8.575
CFB700	10.53 ± 0.08a	6.65 ± 0.28a	70.00 ± 1.41g	0.66 ± 0.007d	0.26 ± 0.009g	0.05 ± 0.011i	0.09 ± 0.002ef	540.63
MCFB700_H2O2_	9.69 ± 0.06d	3.63 ± 0.31cd	75.48 ± 2.43f	0.65 ± 0.002d	0.23 ± 0.007h	0.09 ± 0.010h	0.11 ± 0.013d	537.9
MCFB700_NH3•H2O_	10.20 ± 0.06c	3.23 ± 0.10e	71.94 ± 1.40fg	0.69 ± 0.005a	0.21 ± 0.003i	0.08 ± 0.005h	0.08 ± 0.009f	552.79
MCFB700_HNO3_	3.22 ± 0.05k	1.37 ± 0.08h	75.88 ± 1.42f	0.24 ± 0.001h	0.43 ± 0.003d	0.29 ± 0.008e	0.06 ± 0.006g	514.16

Notes: Results are means ± SD (*n* = 3). Different lowercase letters in the same column indicate significant differences between biochar treatments (*p* < 0.05). The numbers 300, 500, and 700 in the sample ID refer to the pyrolysis temperature 300, 500, and 700 °C, while the subscript, H_2_O_2_, NH_3_•H_2_O, and HNO_3_ refer to hydrogen peroxide, ammonia, and nitric acid-modified biochar, respectively

The number of basic functional groups, pH, CEC, and the SSA of CFBs increased with the pyrolysis temperature (from 300 to 500 °C). At a pyrolysis temperature of 700 °C, the pH and the number of basic functional groups remained the same, while the CEC decreased and the SSA increased significantly (*P* < 0.05). Increasing pH with increasing temperature was consistent with high amounts of ash residuals, which might be explained by the fact that the main components of ash are salts of alkali and alkaline elements and calcite (Singh et al. 2010). On the contrary, the number of acidic functional groups, which is the sum of carboxylic acid, weak acid ester, and phenolic hydroxyl groups, significantly decreased with increasing pyrolysis temperature. Similar findings of decreasing numbers of surface acidic groups with increasing pyrolysis temperature were previously reported and explained by complete combustion and aromatization of the biomass at higher temperatures (Al-Wabel et al. 2013).

The ash content, pH, and number of basic groups of biochars modified with nitric acid (300_HNO3_, 500_HNO3_, and 700_HNO3_) were significantly reduced. This was due to the fact that nitric acid dissolved the ash content in the biochar. On the contrary, the CEC, the number of carboxylic acid, and the weak acid ester groups of biochars modified with nitric acid significantly increased (Table 1). The CEC of 300_HNO3_ biochar increased nearly four times compared to that of the control. The oxidized functional groups on biochar particles could lead to a high CEC and charge density retaining cations as also described elsewhere (Liang et al. 2006). Similar results were found for rice straw biochars under a range of pyrolysis temperatures (Wu et al. 2012).

The SSA of the biochars treated with ammonia was significantly higher than that of the control biochars, in particular for those produced at 300 °C, where the SSA was found to be two times higher than the control. The properties of the MCFBs pyrolyzed at 700 °C did not change with the exception of the pH, the number of alkaline functional groups, and the SSA of the biochar modified with nitric acid, which decreased.

Our results showed that the physico-chemical properties of biochars produced at high pyrolysis temperatures are difficult to change through chemical modification in contrast to those produced at low pyrolysis temperature. This could be associated with the increased formation of aromatic C structures on biochar with increasing pyrolysis temperature (Kim et al. 2011). It is worth mentioning that chemical modification of biochars produced at low temperatures could be used to endow biochars with surface properties that enhance the removal of heavy metals from wastewater, such as, for example, Pb in this study. The development of such tailored or engineered biochars for heavy metal removal from aqueous environments is a promising technique.

The results of the elemental analysis are presented in Table 2. The elemental analysis showed that only a small amount of N (1.05–2.60 wt%) was detected in the modified biochars. The N content of CFBs did not change significantly, while the H and O contents of CFBs increased and the C content decreased significantly with increasing pyrolysis temperature. This resulted in a progressive decrease of the H/C and O/C ratios in the CFBs. These changes were consistent with previous studies on biochars made of rice straw (Wu et al. 2012) and shells of peanuts and coconut coir (Shen et al. 2012). The decrease in the H/C and O/C ratios with increasing pyrolysis temperature can be explained by progressive dehydration, pyrolysis, decarboxylation reactions, and formation of structures containing condensed C (Kim et al. 2011). This is also in agreement with the FTIR data, where we observed that the hydroxyl (−OH; 3600–3200 cm^−1^), alkene (1660–1580 cm^−1^), and anhydride (1300–1199 cm^−1^) functional groups decreased with increasing pyrolysis temperature (see FTIR results below for further discussion).

**Table 2:** The elemental analysis of the coconut fiber-derived biochars (CFBs) and the chemically modified coconut fiber-derived biochars (MCFBs) used in this study

Sample ID	Component,%				Atomic ratio	
	N	C	H	O	H/C	O/C
CFB300	1.05	62.22	3.14	33.60	0.61	0.40
MCFB300_H2O2_	1.14	61.30	4.11	33.45	0.80	0.41
MCFB300_NH3•H2O_	2.31	60.32	4.38	32.99	0.87	0.41
MCFB300_HNO3_	2.60	56.23	3.93	37.24	0.84	0.50
CFB500	1.10	75.99	2.91	20.01	0.46	0.20
MCFB500_H2O2_	1.14	66.95	2.49	29.41	0.45	0.33
MCFB500_NH3•H2O_	1.70	71.23	3.31	23.75	0.56	0.25
MCFB500_HNO3_	2.04	67.28	2.90	27.78	0.52	0.31
CFB700	1.05	71.96	2.60	24.39	0.43	0.25
MCFB700_H2O2_	1.12	72.68	2.96	23.24	0.49	0.24
MCFB700_NH3•H2O_	1.43	73.84	2.66	22.07	0.43	0.22
MCFB700_HNO3_	1.45	72.31	2.47	23.77	0.41	0.25

Notes: Results are means ± SD (*n* = 3). Different lowercase letters in the same column indicate significant differences between biochar treatments (*P* < 0.05). The numbers 300, 500, and 700 in the sample ID refer to the pyrolysis temperature 300, 500, and 700 °C, while the subscript, H_2_O_2_, NH_3_•H_2_O, and HNO_3_ refer to hydrogen peroxide, ammonia, and nitric acid-modified biochar, respectively

The ammonia and nitric acid modifications augmented the contents of N in the MCFBs compared to the CFBs produced at different temperatures. Results of the CFBs pyrolyzed at 300 °C showed decreasing C contents, while the contents of H, O, and N increased for the biochars modified with nitric acid. In the modification process, a large number of bubbles were produced (qualitative observation) on the surface of the biochars, which could possibly be due to the release of carbon dioxide, which would have decreased the C contents. The biochar modifications with ammonia or hydrogen peroxide increased the content of H but the contents of C and O did not change. Therefore, the H/C ratios for all MCFBs and the O/C ratios for all MCFBs_HNO3_ increased. The atomic O/C ratios of the MCFBs500, modified with hydrogen peroxide, nitric acid, and ammonia increased significantly. However, there were no apparent changes for the atomic element ratios of the MCFBs pyrolyzed at 700 °C (Table 2). As pyrolysis progressed, O and H were removed leaving the remaining C to form aromatic C bonds (Kim et al. 2011).

The SEM micrographs of the MCFBs are presented in Fig. S1. Honeycomb-like micropores were observed on both CFBs and MCFBs surfaces. Only minimal morphological differences were detected between the modified and unmodified biochars based on SEM images.

### FTIR analysis for characterization of surface functional groups

**Fig. 1:**
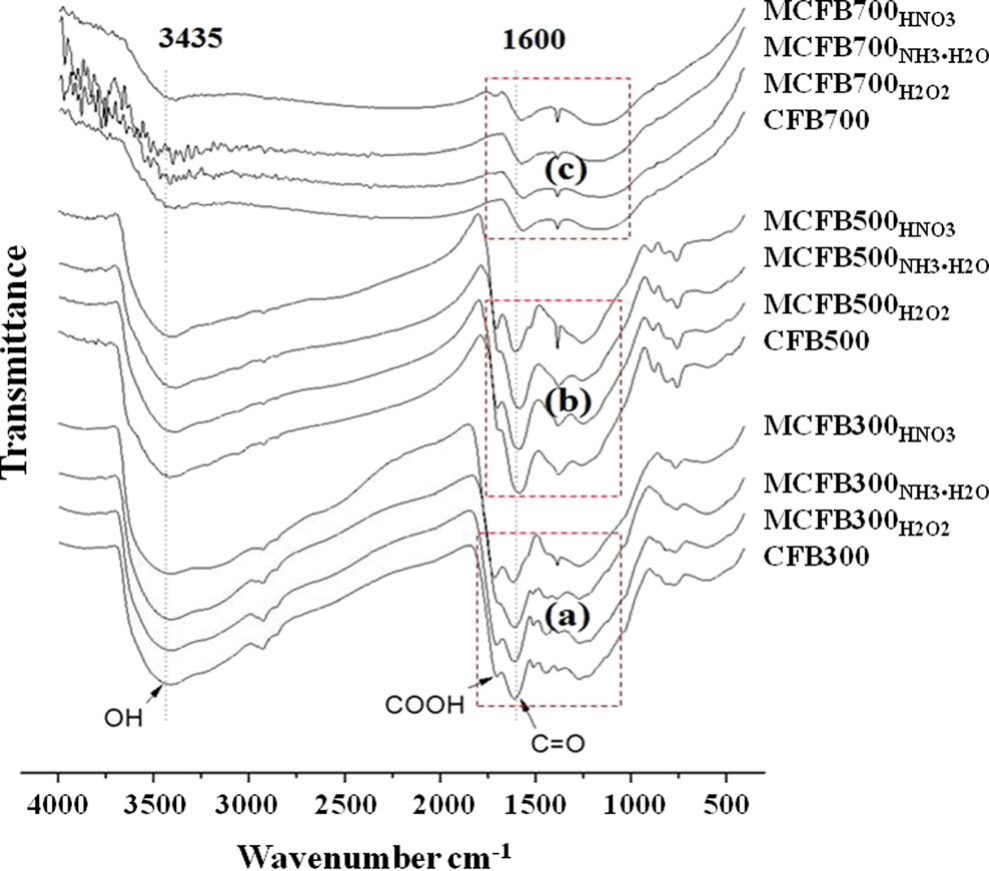
FTIR analysis results of coconut fiber-derived biochars (CFBs) and chemically modified coconut fiber-derived biochars (MCFBs). The numbers of 300, 500, and 700 in the sample ID refer to the pyrolysis temperature 300, 500, and 700 °C, while the subscript, H_2_O_2_, NH_3_•H_2_O, and HNO_3_ refer to hydrogen peroxide, ammonia, and nitric acid-modified biochar, respectively

As SEM did not detect any differences in the micromorphological composition of the CFBs and MCFBs, we used FTIR spectroscopy to determine the biochemical composition of the surface of Pb-sorbed and unsorbed CFBs and MCFBs (Fig. 1, S2; Table S2). The number of peaks and the peak intensity of functional groups on biochars including the peak intensity of hydroxyl O − H stretching (3600–3200 cm^−1^), alkene C = C stretching vibration (1660–1580 cm^−1^), and anhydride C − O − C stretching vibration (1300–1199 cm^−1^) decreased with increasing pyrolysis temperature (Fig. 1). This is in agreement with results of Singh et al. (2012) who reported that the number of functional groups decreased and the degree of condensation of aromatic C increased with increasing pyrolysis temperature.

The most obvious change in the FTIR spectra of biochars produced at 300 °C and modified with ammonia is the decrease in peak intensity to 1705 cm^−1^ due to C = O stretching of carboxyl groups. The amidation reaction of carboxylic acid (−COOH) and ammonia occurred when the solution was heated to about 60 °C, and the yield of amide increased when adding lipase catalysis (Litjens et al. 1999). Both the modified biochars produced at 500 and 700 °C had similar changes in functional groups (Fig. S2b, c), but these changes were not as obvious as those observed for the biochars produced at 300 °C (Fig. S2a).

It seems that at high pyrolysis temperature, the biochars were more stable than those produced at low pyrolysis temperature. It was reported that the chemical stability of the C skeleton was stronger for biochar pyrolyzed at high temperatures (Singh et al. 2012). Our findings are consistent with other studies indicating an increased tendency of aromaticity and aromatic condensation of biochars with increasing pyrolysis temperature (Nguyen et al. 2010).

### Sorption capacity of CFBs and MCFBs for Pb

**Fig. 2:**
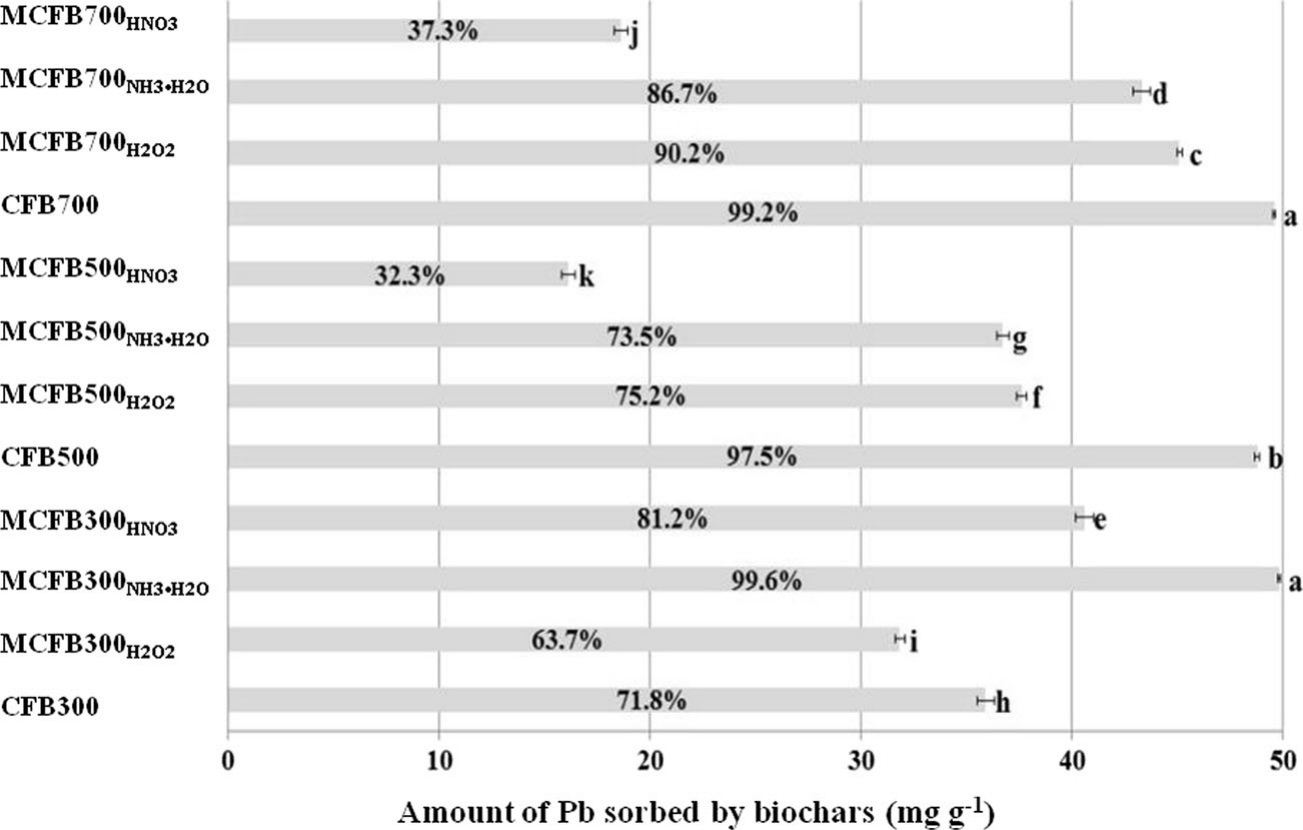
Sorption amount and removal rate of lead (Pb) by coconut fiber-derived biochars (CFBs) and chemically modified coconut fiber-derived biochars (MCFBs). The numbers of 300, 500, and 700 in the sample ID refer to the pyrolysis temperature 300, 500, and 700 °C, while the subscript, H_2_O_2_, NH_3_•H_2_O, and HNO_3_ refer to hydrogen peroxide, ammonia, and nitric acid-modified biochar, respectively

Removal rates of Pb by CFBs and MCFBs from the simulated wastewater (100 mg L^−1^ Pb aqueous solution at pH ) are shown in Fig. 2. We found a significant difference in the amounts of Pb removed from the initial solution among all biochars, The MCFBs_NH3_300 and MCFBs_HNO3_300 increased the capacity of the biochar to remove Pb from the aqueous solution, while all modification treatments of biochars pyrolyzed at 500 and 700 °C decreased the capacity of the biochar to remove Pb. This was due to the changes in the basic properties of the biochars, especially the functional groups. Similarly, Shukla and Pai (2005) reported that the high uptake of heavy metal by oxidized jute could be explained by the generation of –COOH (a weak cationic ion-exchanger). Removal efficiency of Pb by MCFBs_NH3_300 and CFBs700 was found to be 99.6 and 99.2 % of the total Pb in solution, respectively. The Pb concentration in the simulated wastewater was reduced below the current Chinese wastewater discharge standard for Pb (1 mg L^−1^, GB8978—1996, Standard 1996) in water.

## Conclusions

The chemical modifications of CFBs with ammonia, nitric acid, and hydrogen peroxide changed the element composition, functional groups, the CEC, and SSA of the biochars. The increased capacity of the MCFBs300 to remove Pb from aqueous environments compared to the control was probably caused by the changes of biochar properties, such as increased CECs and specific surface areas, multiple functional groups, and activated surface structures. However, it was found that resistance of biochars to chemical treatment increased with pyrolysis temperature. As the CFBs and MCFBs showed promising physico-chemical properties, further studies will be conducted to quantify the adsorption capacities of these CFBs and MCFBs for a range of contaminants.

## Electronic supplementary material

ESM 1 (DOCX 1030 kb)
