# Supplementary material for: Influence of pyrolysis temperature on lead immobilization by chemically modified coconut fiber-derived biochars in aqueous environments
Source: Environ Sci Pollut Res Int. 2016 Aug 29;23(22):22890–6. doi: 10.1007/s11356-016-7428-0 (PMC5101285; doi:10.1007/s11356-016-7428-0)
Supplement: Supplementary file 1 — (DOCX 1030 kb) [file 11356_2016_7428_MOESM1_ESM.docx]

**Influence of pyrolysis temperature on lead immobilization by chemically modified coconut fiber-derived biochars in aqueous environments**

**Weidong Wu^a,b^, Jianhong Li^a^, Nabeel Khan Niazi^c^, Karin Müller^d^, Yingchao Chu^a^, Lingling Zhang^a^, Guodong Yuan^e^, Kouping Lu^b^, Zhaoliang Song^b^, Hailong Wang^b,e*^**

^a^ Ministry of Education Key Laboratory of Protection and Development Utilization of Tropical Crop Germplasm Resources, Hainan University, Haikou 570228, China

^b^ Key Laboratory of Soil Contamination Bioremediation of Zhejiang Province, Zhejiang A & F University, Lin’an, Hangzhou 311300, China

^c^ Institute of Soil and Environmental Sciences, University of Agriculture Faisalabad, Faisalabad 38040, Pakistan

^d^ The New Zealand Institute for Plant & Food Research Limited, Ruakura Research Centre, Private Bag 3123, Hamilton 3240, New Zealand

^e^ Guangdong Dazhong Agriculture Science Co. Ltd., Hongmei Town, Dongguan City, Guangdong 523169, China

^*^ Corresponding author. E-mail addresses: [nzhailongwang@gmail.com](mailto:nzhailongwang@gmail.com); hailong@zafu.edu.cn.


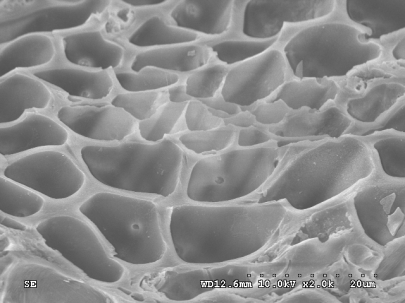

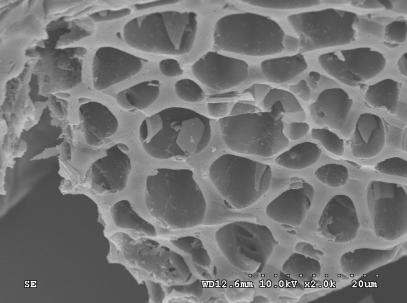

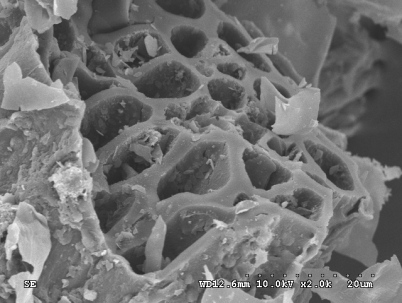


(CFB300) (CFB500) (CFB700)


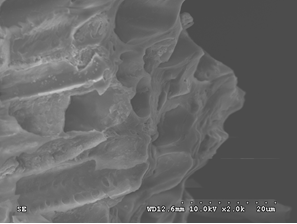

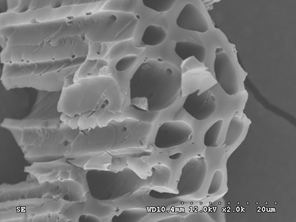

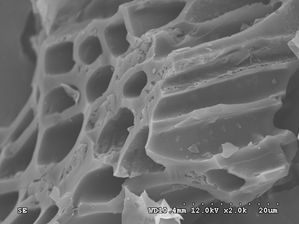


(MCFB300_NH3•H2O_) (MCFB500_NH3•H2O_) (MCFB700_NH3•H2O_)


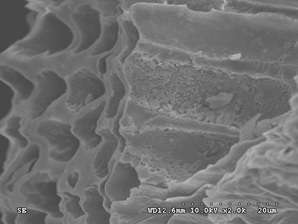

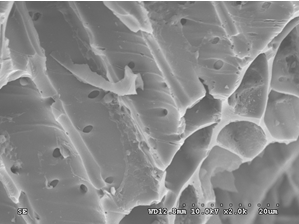

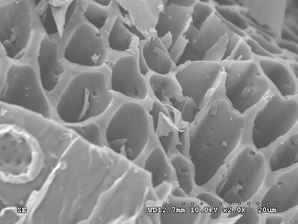


(MCFB300_HNO3_) (MCFB500_HNO3_) (MCFB700_HNO3_)


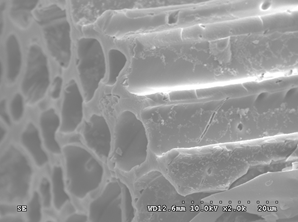

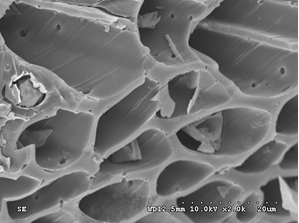

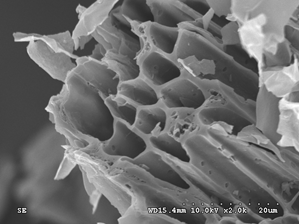


(MCFB300_H2O2_) (MCFB500_H2O2_) (MCFB700_H2O2_)

**Fig. S1** Scanning electron microscope images of different coconut fiber-derived biochars (CFBs) and chemically modified coconut fiber-derived biochars (MCFBs). The numbers of 300, 500 and 700 in the sample ID refer to the pyrolysis temperature 300, 500 and 700°C, while the subscript, H_2_O_2_, NH_3_•H_2_O and HNO_3_ refer to hydrogen peroxide, ammonia and nitric acid modified biochar, respectively

**FTIR analysis for characterization of surface functional groups**

As SEM did not detect any differences in the micromorphological composition of the CFBs and MCFBs, we used FTIR spectroscopy to determine the biochemical composition of the surface of Pb-sorbed and unsorbed CFBs and MCFBs (Fig. S2; Table S1). The number of peaks and the peak intensity of functional groups on biochars including the peak intensity of hydroxyl O−H stretching (3600−3200 cm^−1^), alkene C=C stretching vibration (1660−1580 cm^−1^) and anhydride C−O−C stretching vibration (1300−1199 cm^−1^) decreased with increasing pyrolysis temperature (Fig. S2; Table S1). This is in agreement with results of Singh et al. (2012) who reported that the number of functional groups decreased and the degree of condensation of aromatic C increased with increasing pyrolysis temperature.


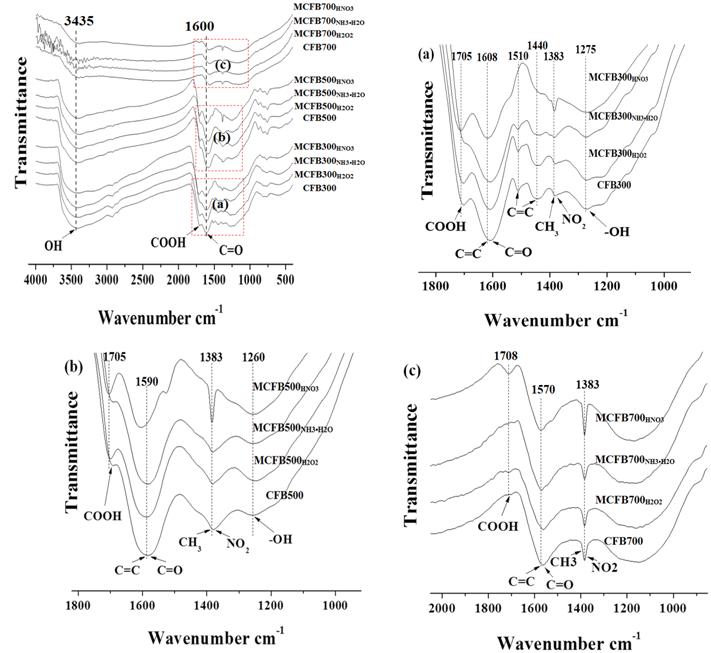


**Fig. S2** FTIR analysis results of coconut fiber-derived biochars (CFBs) and chemically modified coconut fiber-derived biochars (MCFBs). The numbers of 300, 500 and 700 in the sample ID refer to the pyrolysis temperature 300, 500 and 700°C, while the subscript, H_2_O_2_, NH_3_•H_2_O and HNO_3_ refer to hydrogen peroxide, ammonia and nitric acid modified biochar, respectively

For FTIR spectra of MCFBs300, MCFBs500 and MCFBs700, changes were obvious in the regions labeled (a), (b) and (c) in Fig. S2, respectively. The FTIR spectra of MCFBs300 are presented in Fig. S2 (a). The peak intensity of 1705 cm^-1^ C=O stretching of carboxyl increased while the peak intensity of 1608 cm^-1^ C=O stretching of quinones or ketonic acids decreased both on biochars modified with nitric acid and hydrogen peroxide. The changes on the biochar modified with nitric acid were larger than those on the biochar modified with hydrogen peroxide produced at all three temperatures. The peak intensity of 1510 cm^-1^ and 1440 cm^-1^ C=C stretching vibration decreased, while the peak intensity of 1383 cm^-1^ stretching vibrations of −NO_2_ increased for the biochar modified with nitric acid. The MCFB_H2O2_ pyrolyzed at 300°C did not change these peak intensities compared to the control. This might be explained by the following reactions that might have happened during the modification of the biochars:

 Scheme (1)

 Scheme (2)

 Scheme (3)

 Scheme (4)

In oxidation during intense conditions like the presence of nitric acid or hydrogen peroxide, ketone (Scheme 2) was oxidized to form small molecular carboxylic acid (Wang 2005). The nitration reaction (Scheme 3) was observed when mixing phenols or 4-substituted phenols with nitric acid in low concentrations at 25°C (Wang 2005). However, the oxidation reaction of ketone and nitration of benzene or phenols is usually carried out in the presence of a catalyst.

The most obvious change in the FTIR spectra of biochars produced at 300°C and modified with ammonia is the decrease in peak intensity to 1705 cm^-1^ due to C=O stretching of carboxyl groups. The amidation reaction of carboxylic acid (−COOH) and ammonia occurred when the solution was heated to about 60°C, and the yield of amide increased when adding lipase-catalysis (Litjens et al. 1999).

 Scheme (5)

Both the modified biochars produced at 500 and 700°C had similar changes in functional groups (Fig. 1 (b) and (c)), but these changes were not as obvious as those observed for the biochars produced at 300°C.

It seems that at high pyrolysis temperature the biochars were more stable than those produced at low pyrolysis temperature. Research on manure-based biochars and plant-based biochars found the same phenomenon that, the pyrolysis temperature clearly influenced the content of nonaromatic C and the degree of condensation of aromatic C. Moreover, the chemical stability of the C skeleton was stronger for biochar pyrolyzed at high temperatures (Singh et al. 2012). Our findings are consistent with other studies indicating an increased tendency of aromaticity and aromatic condensation of biochars with increasing pyrolysis temperature (Nguyen et al. 2010).

**Table S1** Summary of the main FTIR chemical bond vibrations used in previous studies

| Chemical compound | Chemical bond vibrations | Wavenumber (cm^-1^) | Reference |
| --- | --- | --- | --- |
|  | OH stretching vibration | 3435(3200−3600) | Guo and Bustin 1998 |
|  | C=O stretching of carboxyl and ketones | 1700−1740 | Wu et al. 2012 |
| Aromatic groups | C=O stretching in quinones and ketonic acids or C=C aromatic components | 1600−1630 | Guo and Bustin 1998 |
|  | C=C stretching vibration | 1450−1600 | Moreno-castilla et al. 2000 |
|  | C=C stretching of aromatic C or C−Hx bending vibrations | 1440 | Moreno-castilla et al. 2000 |
|  | Stretching vibrations of −NO_2_ | 1383(1375−1385) | Akhter et al. 1984 |
| Aliphatic groups | Aliphatic deformation of CH_2_ or CH_3_ groups or O−H bending of phenolic−OH | 1375 | Wu et al. 2012 |
| Aromatic groups | Aromatic C−O−structures or phenolic −OH stretching or –OH of carboxyl | 1175−1340 | Guo and Bustin 1998 |

**References**

Akhter MS, Chughtai AR, Smith DM (1984) Reaction of hexane soot with NO_2_/N_2_O_4_. J Phys Chem 88:5334–5342

Guo Y, Bustin RM (1998) FTIR spectroscopy and reflectance of modern charcoals and fungal decayed woods: implications for studies of inertinite in coals. Int J Coal Geolo 37:29–53

Litjens MJ, Straathof Adrie JJ, Jongejan JA, Heijnen JJ (1999) Exploration of lipase-catalyzed direct amidation of free carboxylic acids with ammonia in organic solvents. Tetrahedron 55:12411–12418

Moreno-castilla C, López-Ramón MV, Carrasco-Marín F (2000) Changes in surface chemistry of activated carbons by wet oxidation. Carbon 38:1995–2001

Nguyen BT, Lehmann J, Hockaday WC, Joseph S, Masiello CA (2010) Temperature sensitivity of black carbon decomposition and oxidation. Environ Sci Technol 44:3324–3331

Singh BP, Cowie AL, Smernik RJ (2012) Biochar carbon stability in a clayey soil as a function of feedstock and pyrolysis temperature. Environ Sci Technol 46:11770–11778

Wang X (2005) Organic Chemistry, fourth ed. Higher Education Press of China, Beijing

Wu W, Yang M, Feng Q, McGrouther K, Wang H, Lu HH, Chen YX (2012) Chemical characterization of rice straw-derived biochar for soil amendment. Biomass Bioenergy 47:268–276
